# Cultivating resilience: assessing commercial strawberry cultivars for chilli thrips management in Florida strawberries

**DOI:** 10.1093/jee/toaf041

**Published:** 2025-03-03

**Authors:** Lovely Adhikary, Hugh A Smith, Vance M Whitaker, Sriyanka Lahiri

**Affiliations:** Gulf Coast Research and Education Center, Institute of Food and Agricultural Sciences, University of Florida, Wimauma, FL, USA; Gulf Coast Research and Education Center, Institute of Food and Agricultural Sciences, University of Florida, Wimauma, FL, USA; Gulf Coast Research and Education Center, Institute of Food and Agricultural Sciences, University of Florida, Wimauma, FL, USA; Gulf Coast Research and Education Center, Institute of Food and Agricultural Sciences, University of Florida, Wimauma, FL, USA

**Keywords:** integrated pest management, host plant resistance, cultivar evaluation, strawberry pest management

## Abstract

Strawberry, *Fragaria* x *ananassa* Duchesne (Rosales: Rosaceae), is an important specialty crop in Florida, generating about $500 million in annual revenue. An invasive insect, chilli thrips, *Scirtothrips dorsalis* Hood (Thysanoptera: Thripidae), has emerged as a major strawberry pest, causing considerable yield and revenue loss in recent years. Pesticide application is the leading control option but is not always recommended due to resistance development. Host plant resistance (HPR) can be a novel option to manage *S. dorsalis* sustainably. Four commercial cultivars, ‘Florida Brilliance’, ‘Florida Medallion FL16.30-128’, ‘Sweet Sensation ‘Florida127’, and ‘Florida Pearl FL16.78-109’, were evaluated for their performance in the 2021–2022 field season under the natural population of *S. dorsalis*. In 2022–2023 and 2023–2024, 3 more cultivars, ‘Strawberry Festival’, ‘Florida Radiance’, and ‘Florida Beauty’, were added to this list. Twenty bare-root strawberry transplants were planted in each field plot, and each cultivar was replicated 8 times in a randomized complete block design. Damage on trifoliate, number of adults and larval *S. dorsalis* on trifoliate, number of flowers, and marketable fruit yield were assessed for each cultivar. Results revealed that ‘Florida Pearl 109’ had the highest insect count and damage index of all 3 year. ‘Strawberry Festival’ also showed the same trend after its introduction in the second year. ‘Florida Brilliance’ and ‘Sweet Sensation’ had the lowest damage index, lowest adult insect count, and higher marketable yield compared to ‘Florida Pearl 109’ and ‘Strawberry Festival’. Therefore, utilizing resistant cultivars can be an effective tool for managing *S. dorsalis* in the field.

## Introduction

Strawberry *Fragaria × ananassa* Duchesne (Rosales: Rosaceae) is an important horticultural crop in Florida. The current acreage of strawberries is more than 5,261 ha (13,000 A) with a production value of nearly $500 million (USDA NASS 2023). Strawberry growers in Florida face many challenges associated with strawberry cultivation, including international competition, labor shortage, and unstable market prices. Managing insect pests contributes to these problems because it increases the cost of cultivation (Guan et al. 2018). Many arthropod pests, namely western flower thrips *Frankliniella occidentalis* (Pergande) (Thysanoptera: Thripidae), native flower thrips *Frankliniella bispinosa* Morgan, (Thysanoptera: Thripidae) (Strzyzewski et al. 2021), twospotted spider mite *Tetranychus urticae* Koch (Acari: Tetranychidae), cyclamen mite *Phytonemus pallidus* (Banks) (Acari: Tarsonemidae) (Liburd and Rhodes 2019), tarnished plant bug, *Lygus lineolaris* (Palisot de Beauvois) (Hemiptera: Miridae) (Dumont and Provost 2019), western tarnished plant bug *Lygus hesperus* Knight (Hemiptera: Miridae) (Udayagiri and Welter 2000), and different sap beetle species (Coleoptera: Nitidulidae), *Carpophilus fumatus* Boheman, *Lobiopa insularis* (Castelnau), *Epuraea luteola* Erichson (Rondon et al. 2004) can attack strawberry plants and fruits in field. Among these pests, *T. urticae* is capable of causing almost thirty percent yield reduction in short-day strawberries (Walsh et al. 2002). On the other hand, the thrips species complex was responsible for 60 to 75 percent yield loss in high tunnel strawberry production in Denmark (Nielsen et al. 2021).

*Scritothrips dorsalis* Hood (Thysanoptera: Thripidae) has recently become a major pest in Florida strawberry cultivation (Lahiri et al. 2022). After its reported establishment in the US in 2005 (Hodges et al. 2005), *S. dorsalis* spread to many states, including, Georgia, Alabama, Louisiana, Texas, and New York (Kumar et al. 2023). *Scirtothrips dorsalis* have piercing-sucking mouthparts through which they puncture the plant epidermal cell and feed on the cell contents, leading to cell or tissue necrosis. Heavy infestation can cause leaf brittleness and upward curling of the leaves, ultimately resulting in yield loss (Kumar et al. 2013). In strawberries, the feeding damage includes crinkled and malformed leaves, bronzed and cracked fruits, dwarfed, and stunted plants. The current management program of *S. dorsalis* in conventional strawberry farms heavily depends on synthetic or semi-synthetic insecticides (Renkema et al. 2020). However, broad-spectrum insecticides, such as acetamiprid, spinetoram, can be detrimental to beneficial organisms, including pollinators and predators (Larson et al. 2013, Fernandes et al. 2016, Dale and Borden 2018), and create resistance among the insect population (Vanisree et al. 2011). Therefore, an additional integrated approach is needed to control *S. dorsalis* in strawberries.

Host plant resistance (HPR) can be incorporated into the integrated management of *S. dorsalis* because resistant cultivars can influence the feeding, oviposition, and larval development of the insect pest (Stout and Davis 2009). Resistant cultivars can also be compatible with other pest management techniques within integrated pest management (IPM). For example, resistant plants can be compatible with both chemical and biological control, whereas biological control may not be compatible with chemical control (Teetes 1996). HPR can also be particularly well-suited for organic strawberry cultivation. Host plant characters that are important for insect resistance include plant morphological structures, such as trichomes (Abdelmaksoud et al. 2020), waxy leaf cuticles (Stevenson et al. 1993), and plant chemical compositions such as secondary metabolites (Macel et al. 2019), and volatiles (Bac-Molenaar et al. 2019).

The potential of HPR to reduce damage by twospotted spider mite *T. urticae* on strawberries has been demonstrated in Poland (Łabanowska 2007). Host plant characteristics, such as early flowering and high fruit productivity, reduced feeding damage by tarnished plant bugs *L. lineolaris* on strawberries because the ovipositing females preferred to lay more eggs on the cultivar with fewer receptacles (Rhainds and English-Loeb 2003). In addition, differences in feeding and oviposition preferences were identified among 3 strawberry cultivars that can be utilized for the management of western flower thrips *Frankliniella occidentalis* (Rahman et al. 2010). However, strawberry cultivars have not been evaluated to determine varietal differences in susceptibility to *S. dorsalis* damage. Therefore, in this study, the objective was to evaluate the performance of the commercial strawberry cultivars against *S. dorsalis* under field conditions. The null hypothesis was that there would be no difference in feeding damage injury, insect count, and fruit yield among the cultivars. The alternate hypothesis was that the resistant cultivar would have less foliar damage, lower insect count, and higher yield than other cultivars tested.

## Materials and Method

The study was conducted at the Gulf Coast Research and Education Center (27°45′43′′N; 82°13′38′′W) in Balm, FL, for 3 consecutive strawberry seasons 2021-22, 2022-23, 2023-24. Bare root strawberry transplants were procured from nurseries (G. W. Allen Nursery Ltd., Centreville, Nova Scotia, Canada, and Crown Nursery LLC. Red Bluff, CA, USA).

Four commercial strawberry cultivars, ‘Florida Brilliance’ (U.S. Patent PP30,564, Whitaker et al. 2019), ‘Sweet Sensation Florida127’ (U.S. Patent PP25,574, Whitaker et al. 2015) referred as ‘Sweet Sensation’ hereafter, ‘Florida Medallion FL16.30-128’ (U.S Patent PP33,451) referred as ‘Florida Medallion’ hereafter, and ‘Florida Pearl FL16.78-109’ (U.S. Patent PP 33,477, Whitaker et al. 2023) referred as ‘Florida Pearl 109’ hereafter, were assessed for host plant resistance traits during 2021–2022 season. In 2022–2023, 3 additional commercial strawberry cultivars namely, ‘Florida Radiance’ (U.S. Whitaker et al. 2013, Patent PP 20363) ‘Strawberry Festival’ (U.S. Patent PP14,739, Chandler et al. 2000), and ‘Florida Beauty’ (U.S. Patent PP30,385, Whitaker et al. 2017), were evaluated for the exact attributes. All these cultivars were developed by the University of Florida strawberry breeding program.

Transplanting was done on the 13,11, and 13 of October for the 2021, 2022, and 2023 seasons, respectively. Transplants were planted on raised, double-pressed beds. Each bed was treated as a replication for the experiment. The beds were 98 m in length (320ft) and 80 cm wide, 15 cm high at the edge and 18 cm high at the center with a 45 cm aisle in between and covered with impermeable plastic film (Blockade, Berry Plastics, Evansville, IN, USA). The soil was fumigated during bed formation with 1,3-Dichloropropene, a soil fungicide and nematicide (Telone C-35, 358 L per hectare, Dow Agrosciences, Indianapolis, IN, USA). Glyphosate (Roundup, Bayer Corporation, Whippany, NJ) and Pendimethalin (Prowl H2O, BASF Corporation, Florham Park, NJ) were applied as pre-emergence herbicides, and Carfentrazone-ethyl (Aim, FMC Corporation Philadelphia, PA) as post-emergence to prevent any weed emergence. After transplanting, the plants were irrigated by an overhead sprinkler for ten days and then by a drip irrigation system (431 liter/hectare per minute, T-tape Systems International San Diego, CA). Water-soluble fertilizer (5-2-8-N-P-K, Chemical Dynamics Inc., Plant City, FL, USA) at the rate of 1.12 kg N per day per hectare was applied during the growing season. The plants were treated with recommended fungicides, for example, Captan (Dicarboxamide), and Thiram5C (Tetramethylthiurum disulfide) to control the spread of fungal diseases. Also, one preventative application of *Bacillus thuringiensis kurstaki* (Dipel, Valent Biosciences, Libertyville, IL) was applied to avoid infestation of lepidopteran pests.

The plots were arranged in a randomized complete block design with 5 replications in the 2021–2022 season and 8 replications in the 2022–2023 and 2023–2024 seasons. Strawberry cultivars were planted in a checkerboard pattern so that no cultivars were placed next to each other. Twenty plants of each cultivar were planted on a 9 m (30ft) plot, and a 4.5 m (15 ft) buffer was left in between each plot. Two rows of plants were planted on each bed. The space between the two plants was 38 cm within rows and 30 cm between rows. Data collection started after one month of transplanting on November 9, November 29, and November 13 in the 2021, 2022, and 2023 strawberry seasons respectively. Data was collected for feeding damage rating, number of flowers, marketable fruit yield, and *S. dorsalis* adult and larval count from the trifoliate (3 leaves together on a strawberry plant). Plant tissue samples were collected once in 2 wk. Randomly selected young, fully open trifoliates from each plot were collected and stored in plastic sealed bags (Ziplock, SC Johnson, Racine, WI). The tissue samples were brought back to the lab and stored in −20 °C freezer until further processing.

### Adult and Larval Count

The trifoliates were vigorously stirred in a plastic bag containing 70% ethanol (Thermo Fisher Scientific, Hampton, NH) to dislodge all life stages of thrips. Then, the ethanol was transferred to a square-grided Petri Dish, 8 cm × 8 cm in size where each grid was 1.25 cm × 1.25 cm (Prolab Supply, Florida, USA) to count and identify thrips species using taxonomic keys (Cluever and Smith 2017) under a stereomicroscope at 40× magnification (Carl Zeiss, Germany). Both for damage rating and insect count, 5 trifoliates per plot were considered in 2021, and 8 trifoliates per plot were considered in the 2022 and 2023 seasons.

### Damage Rating on Trifoliates

Damage rating on trifoliates from the feeding injury was done on plants from each plot (0 = No damage, 1= < 10% bronzing of trifoliates, 2 = 10 to 30% bronzing of trifoliates, 3 = 31 to 60% bronzing of trifoliates, 4=> 60% bronzing of trifoliates) (Lahiri and Yambisa 2021). The rating 0 to 4 range was converted into percentage damage on leaves to facilitate analysis and discussion.

### Marketable Fruit Yield and Flower Count

Fruits are harvested weekly. Marketable fruits from each plot were weighed in grams on the same day of harvest. The total number of flowers was counted bi-weekly (once in 2 wk) from each plot.

### Data Analysis

Data were analyzed using the SAS OnDemand for Academics web platform (SAS Institute Inc., Cary, NC, USA). A generalized linear mixed model (PROC GLIMMIX) was used to identify the relationship between the cultivars and the thrips number from trifoliates or damage index caused by thrips feeding. In this model, cultivars, and the time (date of collection from November to February) were considered as fixed effects, and repeated measures on the plots were considered as random effects. Mean separation was done using Tukey’s HSD (*P* < 0.05).

## Results

### *S. dorsalis* Adult Count from the Trifoliates

In the case of adult *S. dorsalis* count, in 2021–2022 strawberry season, ‘Florida Pearl 109’ and ‘Florida Medallion’ had the highest number of *S. dorsalis* count, which was significantly higher than both ‘Florida Brilliance’ and ‘Sweet Sensation’ (*F* = 9.59; df = 3, 128; *P* < 0.0001). In 2022–2023 again ‘Florida Pearl 109’ had the highest *S. dorsalis* count, followed by ‘Florida Beauty’ and ‘Strawberry Festival’. On the other hand, ‘Sweet Sensation’ recorded the lowest number of adult *S. dorsalis* counts from the trifoliates (*F* = 8.77; df = 6, 343; *P* < 0.0001). ‘Florida Brilliance’, ‘Florida Medallion’, and ‘Florida Radiance’ also had a significantly lower number of adult thrips than ‘Florida Pearl 109’. In 2023–2024, ‘Strawberry Festival’ and ‘Florida Pearl 109’, had the highest number of *S. dorsalis* on the trifoliates (*F* = 13.60; df = 6, 392; *P* < 0.0001) compared to all other cultivars (Table 1).

**Table 1.** Mean ± SE of ***S. dorsalis* adult count** from the trifoliates of the strawberry cultivars in 2021, 2022, and 2023 seasons (ANOVA, Proc Glimmix followed by Tukey’s HSD, α = 0.05, SAS Institute Inc., Cary, NC). The mean ± SE labeled with same letter do not differ statistically from other cultivars in the same column (P < 0.05).

Cultivar	2021	2022	2023
Florida Brilliance	0.95 ± 0.234 b	3.66 ± 0.0516 bc	2.29 ± 0.248 b
Sweet Sensation	1.3 ± 0.302 b	2.51 ± 0.344 c	1.57 ± 0.259 b
Florida Medallion	2.52 ± 0.683 a	3.53 ± 0.444 bc	2.5 ± 0.345 b
Florida Pearl 109	3.05 ± 0.66 a	6.76 ± 0.970 a	4.35 ± 0.535 a
Florida Radiance	---	4.03 ± 0.674 bc	1.95 ± 0.303 b
Strawberry Festival	---	5.17 ± 0.738 ab	4.93 ± 0.632 a
Florida Beauty	---	5.30 ± 0.691ab	2.68 ± 0.333 b

### *S. dorsalis* Larval Count from the Trifoliates

In 2021–2022 season, the *S*. *dorsalis* larvae count was significantly higher in ‘Florida Medallion’ and ‘Florida Pearl 109’ compared to ‘Florida Brilliance’ and ‘Sweet Sensation’ (*F* = 10.09; df = 3, 128; *P* < 0.0001). In 2022–2023, ‘Florida Pearl 109’ and ‘Florida Beauty’ had the highest larval counts from the trifoliate followed by ‘Florida Medallion’ and ‘Strawberry Festival’ (Table 2). ‘Florida Radiance’ and ‘Sweet Sensation’ recorded a significantly lower number of larvae from the trifoliate (*F* = 8.30; df = 6, 343; *P* < 0.0001). In the final year, ‘Strawberry Festival’ and ‘Florida Pearl 109’ had the highest larval count from the trifoliate followed by ‘Florida Beauty’ and ‘Florida Medallion’. ‘Florida Radiance’ and ‘Sweet Sensation’ had the lowest larval count (*F* = 34.63; df = 6, 392; *P* < 0.0001).

**Table 2.** Mean ± SE of ***S. dorsalis* larval count** from the trifoliates of the strawberry cultivars in 2021, 2022, and 2023 seasons (ANOVA, Proc Glimmix followed by Tukey’s HSD, α = 0.05, SAS Institute Inc., Cary, NC). The mean ± SE labeled with same letter do not differ statistically from other cultivars in the same column (*P* < 0.05).

Cultivars	2021	2022	2023
Florida Brilliance	3.47 ± 0.605 b	5.05 ± 0.867 bc	11.01 ± 1.524 bc
Sweet Sensation	3.9 ± 1.18 b	4.23 ± 0.647 c	7.98 ± 1.06 c
Florida Medallion	8.9 ± 1.66 a	7.89 ± 1.11 ab	14.03 ± 1.23 b
Florida Pearl 109	7.64 ± 1.16 a	8.67 ± 1.6 a	22.328 ± 1.574 a
Florida Radiance	---	4.30 ± 0.677 c	9.64 ± 1.65 c
Strawberry Festival	---	7.44 ± 1.10 ab	22.343 ± 1.53 a
Florida Beauty	---	8.58 ± 1.4 a	14.703 ± 1.85 b

### Damage Rating on the Trifoliates

**Fig. 1.**
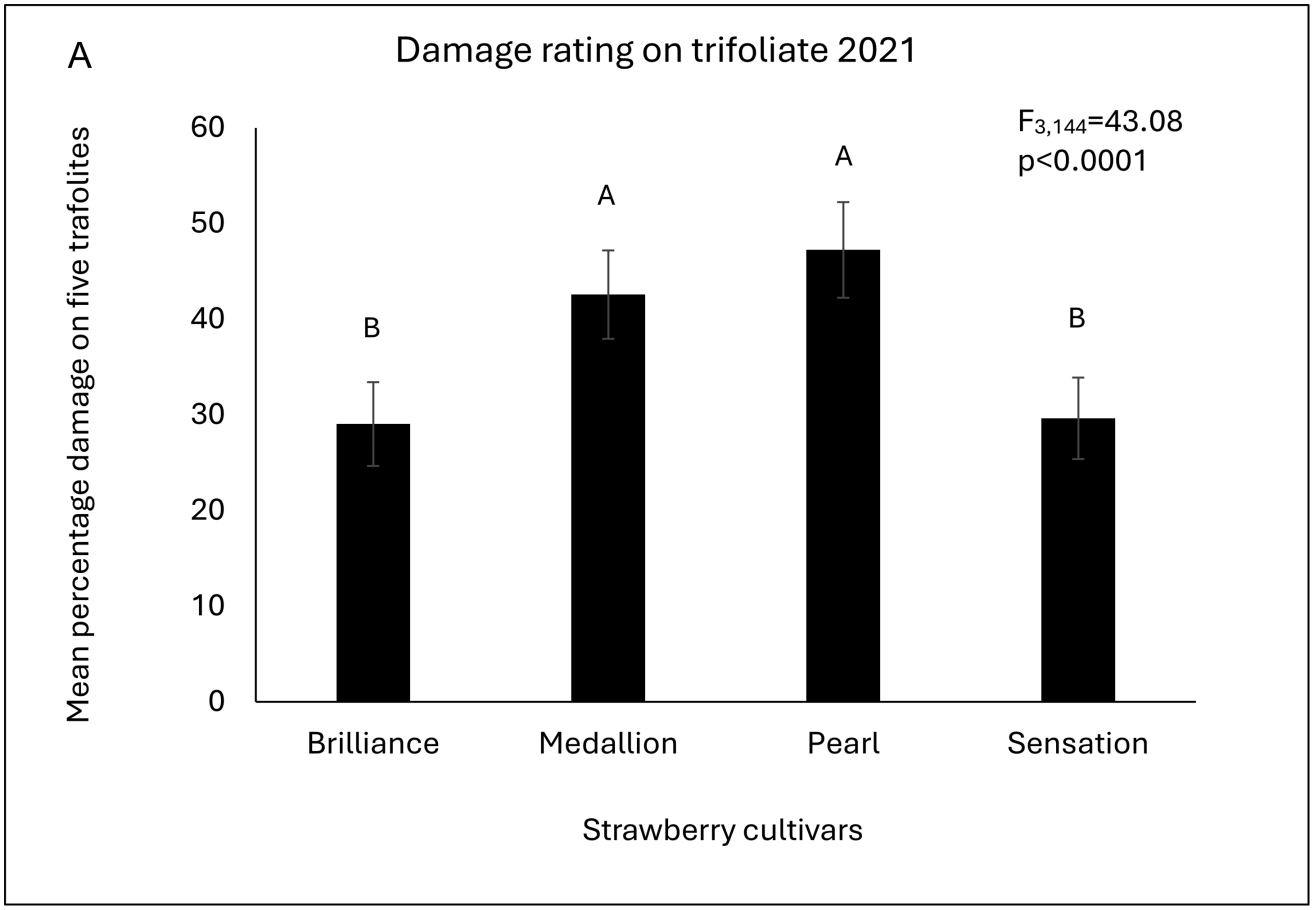

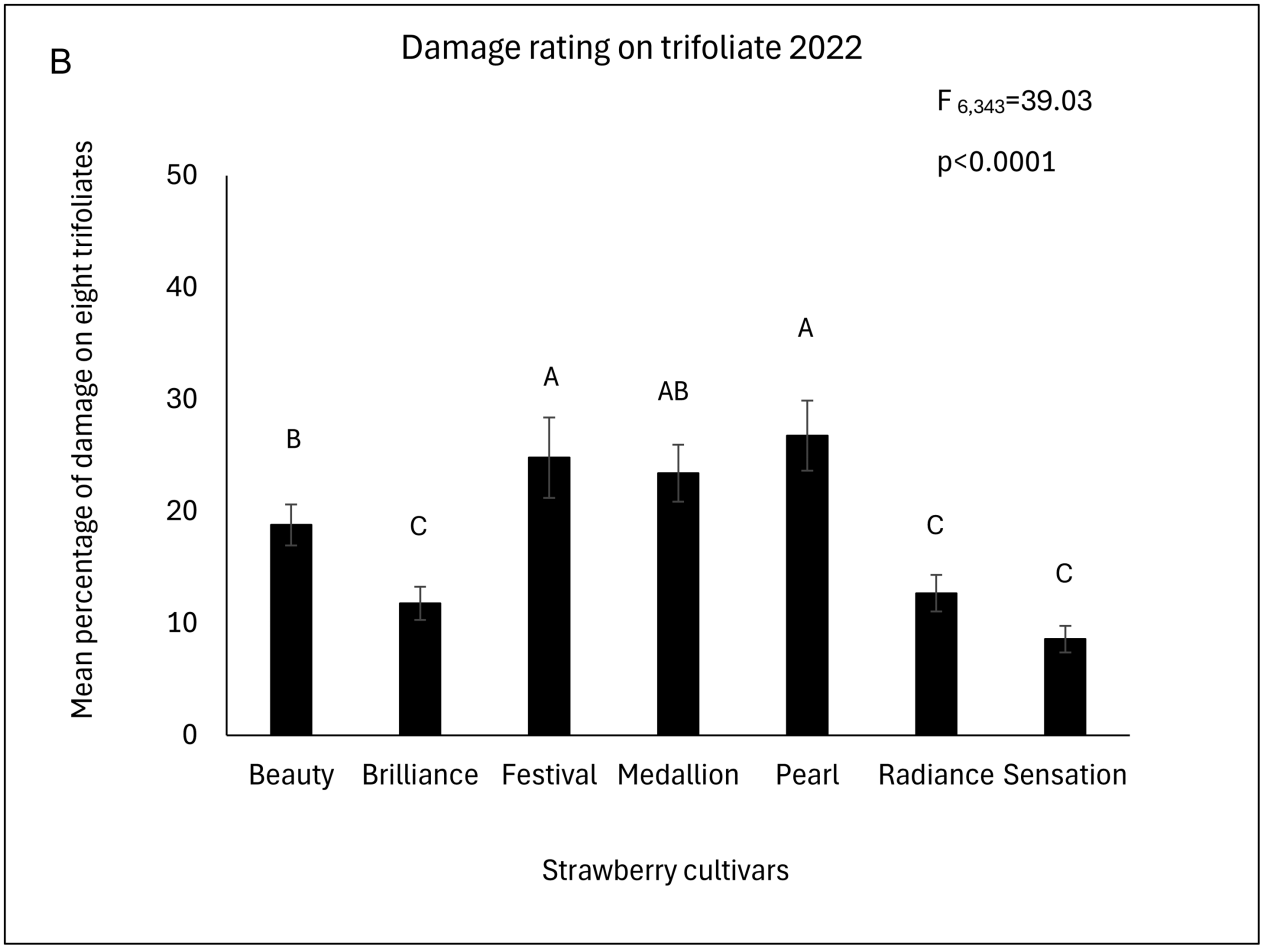

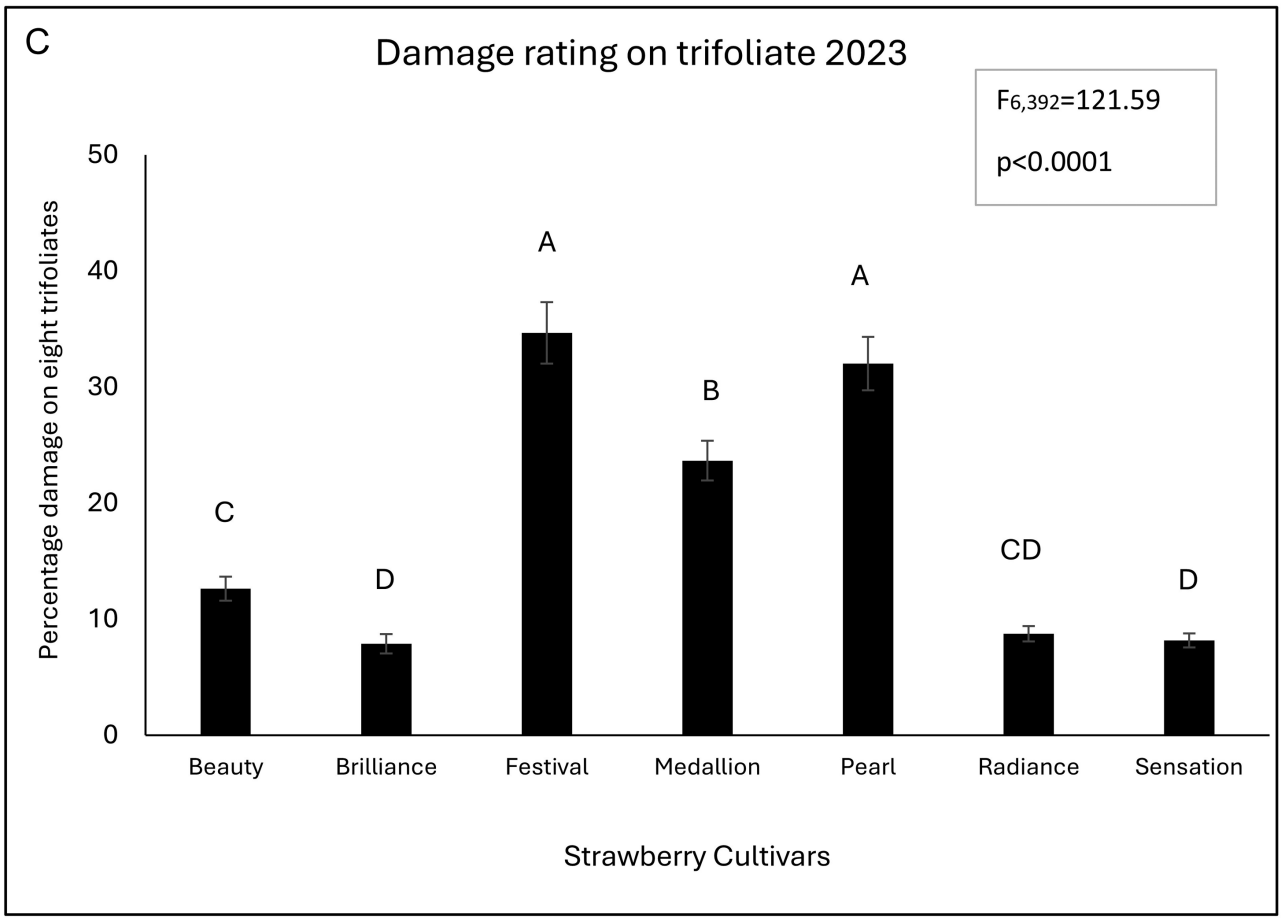
(A) Mean percentage **feeding damage rating** on trifoliate of 4 strawberry cultivars during 2021, strawberry season from the research plots in Balm, FL. Bars with the same letters are not significantly different (PROC Glimmix, Repeated measures, followed by Tukey HSD α = 0.05). (B) Mean percentage **feeding damage rating** on trifoliates during 2022, strawberry season from the research plots in Balm, FL. Bars with the same letters are not significantly different (PROC Glimmix, Repeated measures, Tukey HSD α = 0.05). (C) Mean percentage **feeding damage rating** on trifoliates during 2023 strawberry season from the research plots in Balm, FL. Bars with the same letters are not significantly different (PROC Glimmix, Repeated measures, Tukey HSD α = 0.05).

In the first year (2021), 4 cultivars were tested. The feeding damage injury significantly differed among the cultivars (*F* = 43.08; df = 3, 144; *P* < 0.001). ‘Florida Pearl 109’ and ‘Florida Medallion’ had higher feeding damage on the trifoliates compared to ‘Florida Brilliance’ and ‘Sweet Sensation’ (Fig. 1A). In the following year (2022), 3 more cultivars, ‘Strawberry Festival’, ‘Florida Beauty’, and ‘Florida Radiance’ were added to the test. Among these 7 cultivars, ‘Florida Brilliance’, ‘Sweet Sensation’, and ‘Florida Radiance’ had significantly lower feeding damage injury on the trifoliate compared to the other 4 cultivars (*F* = 39.03; df = 6, 343; *P* < 0.001). ‘Florida Beauty’ sustained lower feeding injury compared to ‘Strawberry Festival’, and ‘Florida Pearl 109’. ‘Strawberry Festival’ and ‘Florida Pearl 109’sustained the highest feeding injury among the 7 cultivars being compared (Fig. 1B).

In the last year (2023), the feeding damage injury trend was similar to 2022. The ‘Strawberry Festival’ and ‘Florida Pearl 109’ recorded the highest feeding damage, followed by ‘Florida Medallion’ which was significantly lower than ‘Strawberry Festival’ and ‘Florida Pearl 109’ but higher than the rest of the cultivars. ‘Florida Beauty’ and ‘Florida Radiance’ had significantly lower damage than the previously mentioned cultivars. The lowest damage was recorded in ‘Florida Brilliance’ and ‘Sweet Sensation’ (*F* = 121.59; df = 6, 392; *P* < 0.0001) (Fig. 1C).

### Flower Count

In the 2021–2022 season, ‘Sweet Sensation’ had fewer flowers than all other cultivars, i.e., ‘Florida Brilliance’, ‘Florida Medallion’, and ‘Florida Pearl 109’ (*F* = 9.59; df = 3, 128; *P* < 0.0001). In the next year, 2022–2023, with 3 new cultivars introduced, the flower count trend changed from the first year (Table 3). The highest number of flowers was recorded on ‘Strawberry Festival’ and ‘Florida Beauty’, followed by ‘Florida Brilliance’ and ‘Florida Radiance’ (*F* = 5.68; df = 6, 343; *P* < 0.0001). The number of flowers on ‘Florida Pearl 109’, ‘Sweet Sensation’, and ‘Florida Medallion’ were significantly lower than ‘Florida Beauty’ and ‘Strawberry Festival’. In 2023–2024, the flower production trend was similar to the previous year. ‘Florida Beauty’ and ‘Strawberry Festival’ produced the highest number of flowers (*F* = 45.90; df = 6, 392; *P* < 0.0001). On the other hand, the ‘Florida Medallion’ produced the lowest number of flowers among all the cultivars.

**Table 3.** Mean ± SE of **flower count** from the trifoliate of the strawberry cultivars in 2021, 2022, and 2023 seasons (ANOVA, Proc Glimmix followed by Tukey’s HSD α = 0.05, SAS Institute Inc., Cary, NC). The mean ± SE labeled with same letter do not differ statistically from other cultivars in the same column (*P* < 0.05).

Cultivars	2021	2022	2023
Florida Brilliance	13.45 ± 0.696 a	9.5 ± 1.02 abc	14.03 ± 1.415 b
Sweet Sensation	9.775 ± 0.864 b	8.25 ± 0.915 bc	11.281 ± 0.99 cd
Florida Medallion	13.225 ± 0.745 a	8.17 ± 0.810 bc	8.25 ± 0.956 e
Florida Pearl 109	13.725 ± 0.709 a	7.83 ± 0.938 c	8.82 ± 0.86 de
Florida Radiance	---	10.07 ± 1.07 ab	11.718 ± 1.15 bc
Strawberry Festival	---	11.125 ± 1.37 a	18.906 ± 2.47 a
Florida Beauty	---	10.35 ± 1.157 ab	17.031 ± 1.63 a

### Marketable Fruit Yield

**Fig. 2.**
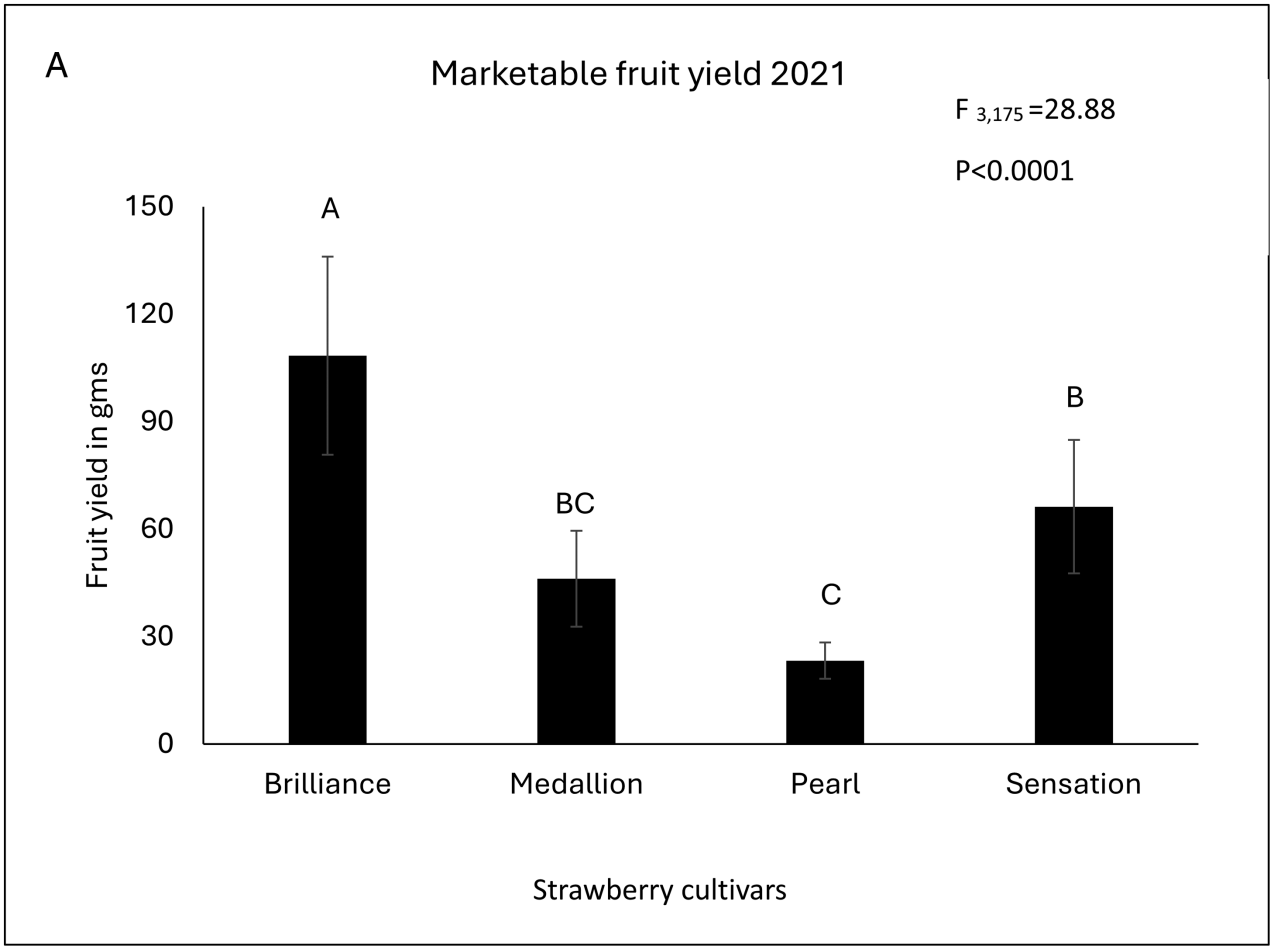

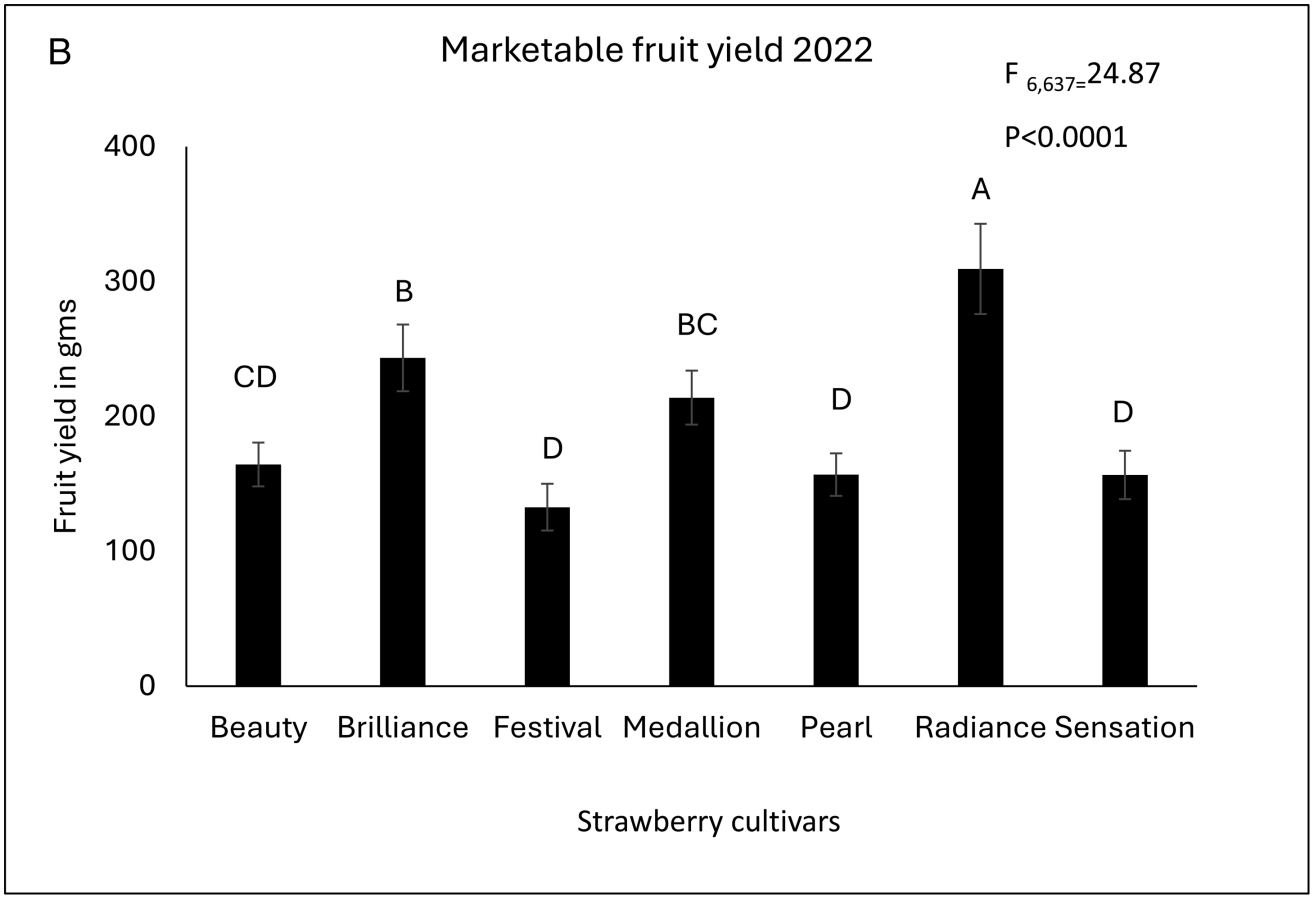

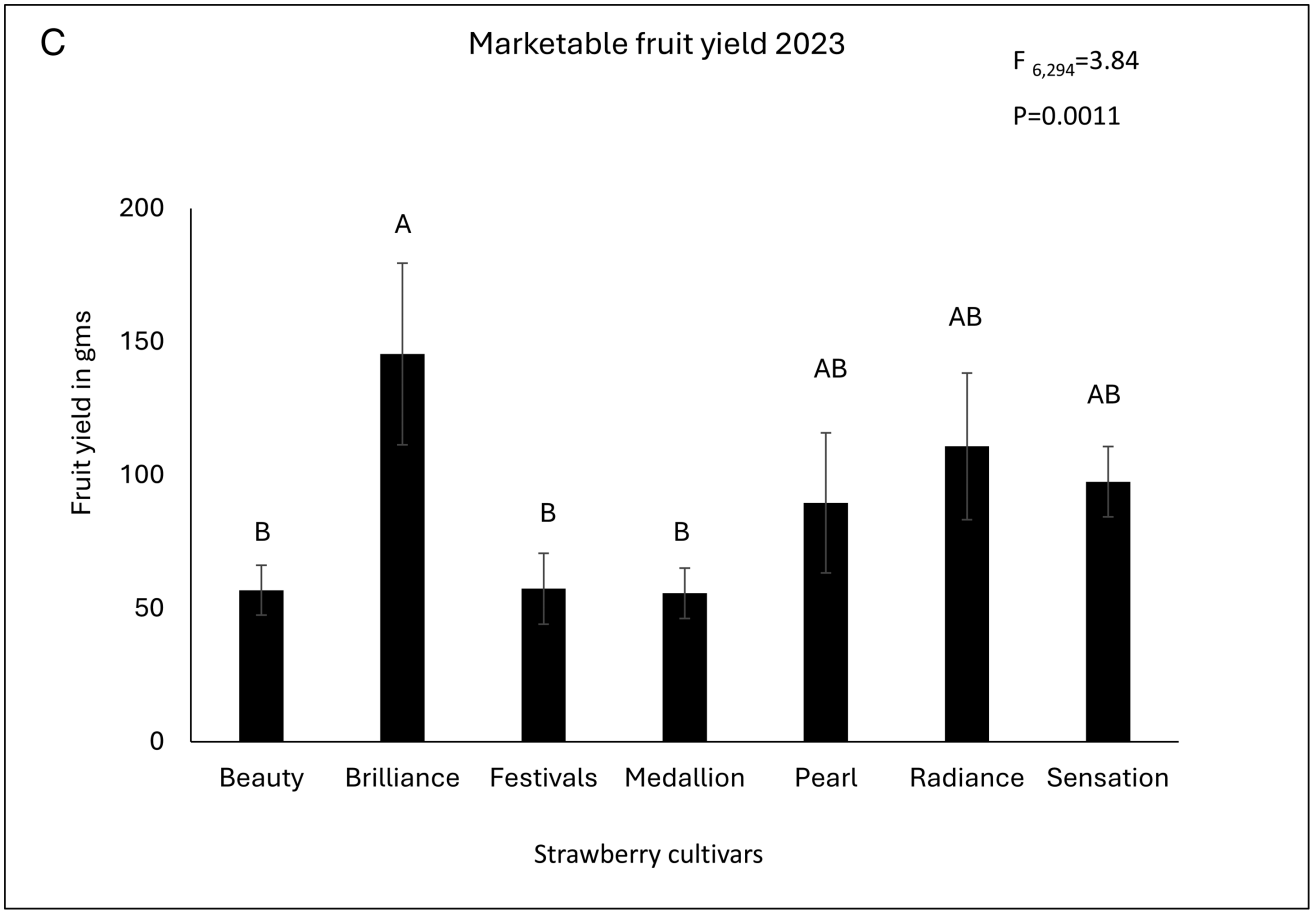
(A) Mean (± SE) **marketable fruit yield** (gms) of strawberries from the different cultivars during 2021 (PROC Glimmix, Repeated measures, Tukey HSD α = 0.05). Bars with the same letters are not statistically different from each other. (B) Mean (± SE) **marketable fruit yield** (gms) of strawberries from the different cultivars during 2022 (PROC Glimmix, Repeated measures, Tukey HSD α = 0.05). Bars with the same letters are not statistically different from each other. (C) Mean (± SE) **marketable fruit yield** (gms) of strawberries from the different cultivars during 2023 (PROC Glimmix, Repeated measures, Tukey HSD α = 0.05). Bars with same letters are not statistically different from each other.

In the first year, 2021–2022, ‘Florida Brilliance’ produced the highest marketable fruit (*F* = 28.88; df = 3, 175; *P* < 0.0001). In contrast, ‘Florida Pearl 109’ produced the lowest fruit yield among all the cultivars. For ‘Sweet Sensation’ and ‘Florida Medallion’ fruit yield lies in the middle; ‘Sweet Sensation’ produced a significantly higher yield than ‘Florida Pearl 109’ (Fig. 2A). In 2022–2023, ‘Florida Radiance’ produced the highest marketable yield, followed by ‘Florida Brilliance’ and ‘Florida Medallion’ (*F* = 24.87; df = 6, 637; *P* < 0.001) (Fig. 2B). In 2023–2024, ‘Florida Brilliance’ produced the highest marketable yield, which is not different from the yield produced by ‘Florida Pearl 109’, ‘Florida Radiance’, and ‘Sweet Sensation’ (*F* = 3.84; df = 6, 294; *P* = 0.0011). ‘Florida Beauty’, ‘Strawberry Festival’, and ‘Florida Medallion’ produced significantly lower yields than ‘Florida Brilliance’ (Fig. 2C).

## Discussion

The result shows the total insect count from the trifoliate differs among the tested strawberry cultivars. *S. dorsalis* are weak fliers (Panthi et al. 2021b) and they start colonizing the field starting from the field borders from the alternate hosts (Kaur et al. 2024). The probability of the number of insects established on each cultivar possibly depends on the initial host selection, the egg hatch percentage, and the rate of larval development. The population increase on these plants depends on the plant’s character or fitness. Better host plant fitness supports the faster development of the immatures and higher survival rates of thrips (Cao et al. 2022).

The egg hatch and larval development on the host plant depend on the composition of secondary metabolites (Maharijaya et al. 2012). For example, a higher concentration of alkenes supports larval development of *F. occidentalis* in pepper (Maharijaya et al. 2012), or a lower amino acid content can reduce the feeding damage on the plant (Mollema and Cole 1996). The cultivars with higher insect count from the trifoliate possibly provided a better microclimate and nutrition for larval development.

The damage index was mainly due to feeding damage on the trifoliate. ‘Florida Pearl 109’ had higher feeding damage to the trifoliate in all 3 yr compared to all other cultivars (Fig. 1A–C). During the 2022–2023 and 2023–2024 strawberry seasons, ‘Strawberry Festival’ produced damage similar to ‘Florida Pearl 109’. The feeding preference on host plants by different thrips species depends on various factors, including the nutritional configuration of the host plant (Scott Brown et al. 2002). Though the analysis of cultivar nutritional composition was beyond the scope of this study, the factors can vary. A previous study with the western flower thrips *F. occidentalis* found that the host plant supporting the larger thrips population had a higher concentration of protein and carbohydrates (Scott Brown et al. 2002). *Scirtothrips dorsalis* prefers to feed on young plant tissue but can feed on all above-ground plant material (Kumar et al. 2013). Feeding and oviposition preference for young tissue over older tissue is usual in herbivores (Badenes-Perez et al. 2014) because the young leaves can contain more nutrition and less toxic metabolites (Raupp et al. 1988). The robust canopy with more leaves in ‘Florida Pearl 109’ and ‘Strawberry Festival’ could have been a potential source for more young tissue for *S. dorsalis* feeding.

The damage index on ‘Florida Pearl 109’ and ‘Strawberry Festival’ can result from various factors, such as plant physical and morphological attributes and nutritional content (Fu et al. 2019). On the other hand, the ‘Florida Brilliance’ and ‘Sweet Sensation’ in all 3 yr, and ‘Florida Radiance’ in the last 2 yr had consistently lower damage index, which was possible if these cultivars did not support larval development unlike their counterparts. Although all the cultivars had some degree of damage and insect population, ‘Strawberry Festival’ and ‘Florida Pearl 109’ emerged as the most suitable cultivars for *S. dorsalis*. However, no study has been done to evaluate the effect of cultivars on the population development of *S. dorsalis*. Other studies suggest that resistant cultivars reduce the reproduction rate and extend the developmental time of *F. occidentalis* on cucumbers (Soria and Mollema 1995). The longer developmental time of thrips immatures may lead to slower population development and less feeding injury on the host plant.

*S. dorsalis* remains active on host plants in the central and south Florida region throughout the year (Panthi et al. 2021a). This insect can develop at a temperature range from 9.7 °C to 33 °C (Tatara 1994; Seal et al. 2010). In Florida, the average temperature during the winter months or strawberry growing period remains well above 9.7 °C (The Florida Automated Weather Network Balm), which is optimum for the growth and development of this pest. As our study was done in open-field conditions, all the cultivars were exposed to the same climate during the growing season. The difference in the number of *S. dorsalis* on each of these cultivars is due to their suitability as a host of this pest.

In this study, the recorded flower number from the cultivars suggests that *S. dorsalis* infestation does not impair the flower production capacity of the strawberry plants. ‘Strawberry Festival’ produced the highest number of flowers during the 2022 and 2023 seasons and had the highest feeding damage index on the leaves, leading to the assumption that flower production depends on environmental and plant physiological conditions (Darnell et al. 2010). Though *S. dorsalis* does not influence flower production, this insect substantially reduces marketable fruit production because it feeds on the ripening fruit, resulting in the bronzing and cracking of the fruit (Panthi and Renkema 2020). Our result shows that the ‘Strawberry Festival’ produced the highest number of flowers during 2022 and 2023 but failed to deliver the expected yield because of the pest pressure. A previous study showed that substantial thrips infestation negatively affects the plant’s leaf size and carbon allocation because the infestation impairs the plant’s photosynthesis ability, eventually reducing the fruit yield (Welter et al. 1990). In our study, ‘Florida Brilliance’ and ‘Florida Radiance’ produced higher marketable fruits than other cultivars. In a recent study, authors found ‘Florida Brilliance’ produced a higher marketable yield despite having higher foliar damage, demonstrating evidence of host plant resistance (Lahiri et al. 2024).

A sustainable management strategy can be achieved by embracing multiple pest control techniques. Host plant resistance, cultural control, and the use of natural enemies are the predominant approaches for any pest control system (Sharma and Ortiz 2002). The cultivars supporting fewer *S. dorsalis* can be incorporated into the integrated management approach. The lower number of thrips would require fewer insecticide applications, eventually reducing the dependency on chemical control (Teetes 1996). Also, a plant with resistant characteristics can have nutritional and morphological factors that can affect the physiology and behavior of the target pest. Alteration in physiology and behavior can lead to a reduction in feeding damage and ultimately lower the need for chemical application (Slansky 1990). Resistance cultivars can reduce the damage on the susceptible cultivars as well when grown together through ‘associational resistance’ (Koski et al. 2021). Also, a resistant genotype can be immune to different groups of insects. For example, the wild strawberry *Fragaria vesca* L. (Rosales: Rosaceae), which is resistant to strawberry leaf beetle *Galerucella tenella* L. (Coleoptera: Chrysomelidae) also found to be resistant to generalist aphids *Aphid gossypii* Glover (Hemiptera: Aphididae) (Musaqaf et al. 2022). Resistant cultivars can influence the effect of biological control by increasing the developmental time which in turn increases the time of exposure of the pest to the natural enemy (Price et al. 1980, Peterson et al. 2016).

Our study suggests ‘Florida Brilliance’, ‘Sweet Sensation’, and ‘Florida Radiance’ are better-suited cultivars for combating *S. dorsalis* in the Florida Strawberry field. Though these cultivars are already commercially released, future plant breeding efforts can be informed through this study. Adopting these cultivars in the field can reduce insecticide application and increase the chances of survival ability of augmentative biocontrol agents.

In summary, our study shows that host plant resistance can be employed as a valuable tool in strawberry pest management, focusing on *S. dorsalis*. Future studies should examine the mechanisms of the HPR documented in this experiment, pivoting on whether the resistance is incurred through antixenosis or antibiosis. Plant physical or morphological characteristics that either deter insects from feeding, ovipositing, or negatively impact the development, survival, and reproduction of the insects should be identified. Incorporating these traits into the study will provide clarity about which specific feature of the strawberry plants is contributing to the resistance. Molecular markers associated with these plant traits from wild strawberry genotypes and breeding lines would allow breeders to select the possible genotypes that can be used for the development of new resistant cultivars.
